# Lessons in leadership: Developing a longitudinal evidence‐based teaching curriculum on professionalism in healthcare

**DOI:** 10.1002/jhrm.70005

**Published:** 2025-04-24

**Authors:** Allen M. Chen

**Affiliations:** ^1^ Department of Radiation Oncology University of California, Irvine, Chao Family Comprehensive Cancer Center Orange California USA

## Abstract

Given the responsibility of healthcare organizations to promote positive workplace cultures, the development of appropriate teaching material focused on professionalism is of relevance. A longitudinal evidence‐based educational curriculum was thus constructed to equip participants with tools to enhance team‐based care and to create an inclusive, respectful environment. Core themes on which to center the curriculum were identified based on the preferred reporting items for systematic review and meta‐analysis protocols (PRISMA‐P) statement. A MEDLINE search was undertaken to identify original peer‐reviewed works using terms associated with professionalism in healthcare with the goal of building a foundational basis. Articles published from January 2014 to January 2024 and restricted to the English language were included. Based on the search results, a 12‐month curriculum designed to promote workforce engagement and discussion was established. The 537 peer‐reviewed publications selected to develop this thematic framework were broadly categorized as follows: ethics/accountability (*N* = 131); conflict resolution (*N* = 120); collaboration (*N* = 107); interpersonal communication (*N* = 70); empathy (*N* = 57); and wellness (*N* = 52). Between November 2023 and November 2024, a total of 12 sessions were scheduled. The feasibility of developing a standardized, evidence‐based curriculum on workplace professionalism was demonstrated. The practical implications are discussed.

## INTRODUCTION

Professionalism arguably serves as the foundation on which healthcare rests. However, definitions for this core competency are elusive and imprecise.^1^, ^2^, ^3^ Too many, professionalism represents an abstract concept that one knows is vital to being a provider and can affect the quality of care, patient outcomes, and even morale in the workplace; yet characterizations of this principle are often subjective, non‐specific, and/or ambiguous. To illustrate, the Accreditation Council of Graduate Medical Education (ACGME) states that proficiency in this domain is “primarily behavioral and attitudinal and is demonstrated as part of all other competency domains.^4^” The Association of American Medical Colleges (AAMC) is even more broad, defining professionalism as a “commitment to carrying out professional responsibilities and an adherence to ethical principles.^5^”

Given the complexities inherently associated with professionalism, education on this subject can thus be fraught with challenges. A lack of teaching material on this subject also leads to difficulty in promoting standards of conduct. While the overarching goals of professionalism training are to create a more inclusive, empathetic, and harmonious environment for both patients and providers, how to do so is uncertain. Indeed, the optimal strategies to bring attention to professionalism in the healthcare setting, to highlight its essentialness, and to integrate practical lessons into formal teaching activities have not been identified. Despite the agreement among stakeholders on the importance of professionalism, questions persist on how to best disseminate the relevant material to augment learning. Furthermore, there is a dearth of literature on how to standardize training. To address these needs, a longitudinal professionalism‐based curriculum consisting of interactive seminars and roundtable discussions was prepared to equip participants with tools to enhance effective care and to create an inclusive learning environment. The purpose of this study was to report a single‐institutional experience with the development of this literature‐based curriculum.

## METHODS AND MATERIALS

The design of this professionalism curriculum originated from an internal grant that was awarded for the purpose of developing an interactive teaching module to raise awareness of professionalism issues, specifically as they relate to inclusion, civility, and conduct in the setting of an expanding workforce. To develop this curriculum, we initially used Kern's 6‐step curriculum development approach.^4^ In preparation for substantive development, themes on which to center the curriculum were identified through an evidence‐based review based on the preferred reporting items for systematic review and meta‐analysis protocols (PRISMA‐P) statement. The initial screen was conducted on September 15, 2023 and repeated again on October 1, 2023, November 1, 2023, December 10, 2023, January 5, 2024, and Feburary 14, 2024.

To start, a MEDLINE literature search of publicly accessible publications was undertaken to identify original peer‐reviewed works pertaining to the topic of professionalism in the healthcare workplace. The search terms “professionalism,” “civility,” “conduct,” “empathy,” “humanism” “inclusion,” “bullying,” “teamwork,” “conflict,” “disruptive,” and “communication” were inputted in various permutations through the MEDLINE database to comprehensively initiate this exercise. To ensure that all possible publications were captured, multiple iterations of the search were processed on different days. Boolean operators were routinely used to combine search terms, and advanced field tags were incorporated to refine the selection process in an attempt to limit the analysis to clinically oriented papers focused on healthcare. Reference lists from included articles were cross‐checked to identify additional articles. Review articles and papers presented as conference proceedings were excluded, as well as those originating from areas outside of medicine. Articles published from January 2014 to January 2024 with full text available and restricted to the English language and human subjects were included. The full bibliographies of identified articles were reviewed, and irrelevant studies and/or those of insufficient quality were selectively removed at the discretion of the investigator. Where individual works were included in multiple published series, the most complete or recent article was cited. To measure quality of the studies, the National Institute of Health's quality‐measure tool for optimizing internal validity was used, and only those studies rated as “good” or “fair” were included in the development of the curriculum^6^. An interpretive synthesis of the available publications was then presented and formed the foundation on which a standardized curriculum was devised with the practical objective of raising professionalism awareness in the workplace.

## RESULTS

### Search results

**FIGURE 1:**
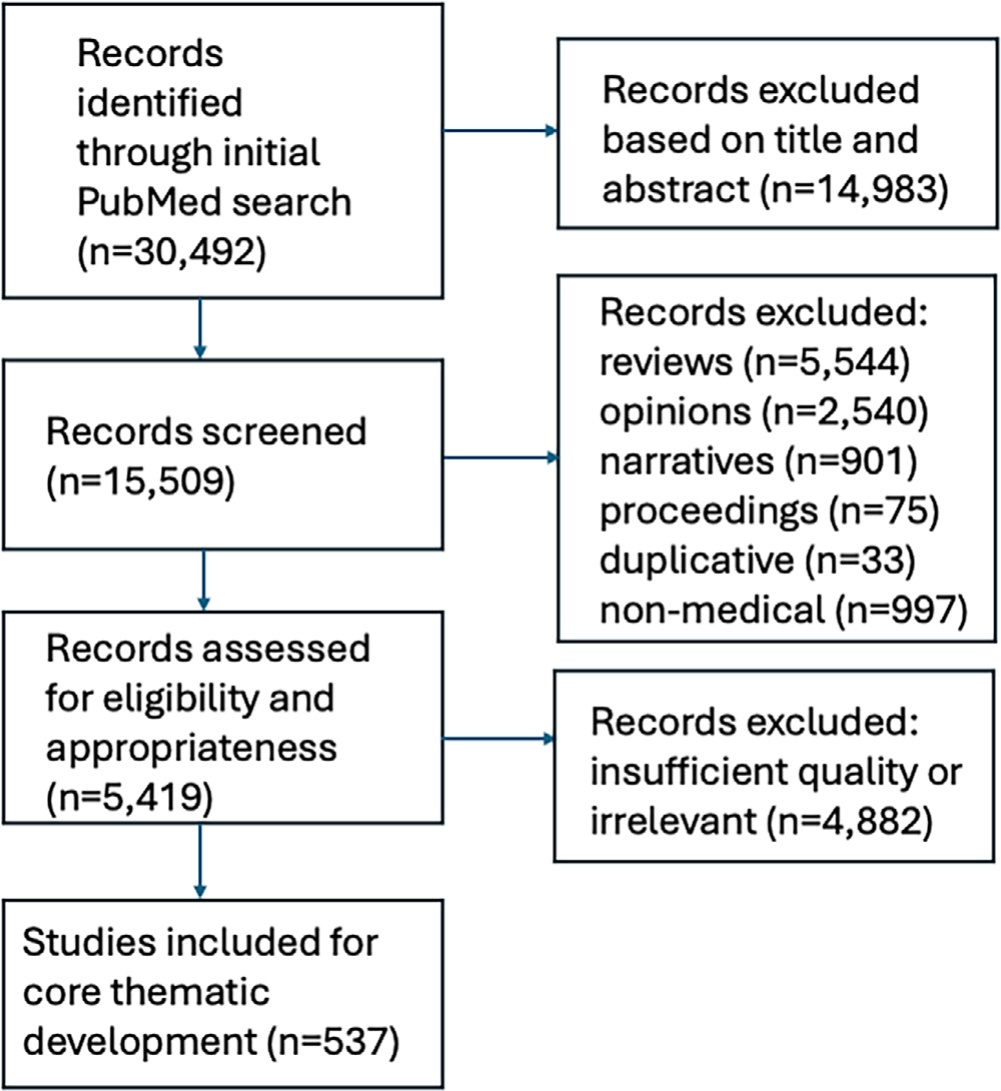
Schematic flow diagram of search process.

The initial search yielded 30,492 independent articles. After cursory screening of these publications based on title and abstract, a total of 15,509 studies proceeded to full‐text screening. Subsequently, 9,093 articles were excluded because they were review articles (*N* = 5,544); opinion pieces, letters, or editorials (*N* = 2,540); narratives (*N* = 901); conference proceedings or position papers (*N* = 75); or duplicative from prior works (*N* = 33). Another 997 publications were excluded because they originated from non‐medically related fields and/or were designed from a perspective outside of mainstream medicine. Publications were included for imputation into the framework for curriculum design if they had a clearly stated and primary curricular objective focused specifically on professionalism in the workplace and were published as an original research piece. Based on insufficient quality and/or relevance along these lines, another 4882 articles were excluded. A total of 537 peer‐reviewed articles thus were included and formed the foundational basis for final curriculum design. A schematic illustration of the flowchart outlining the results of the search strategy is shown in Figure 1.

### Curriculum design

**FIGURE 2:**
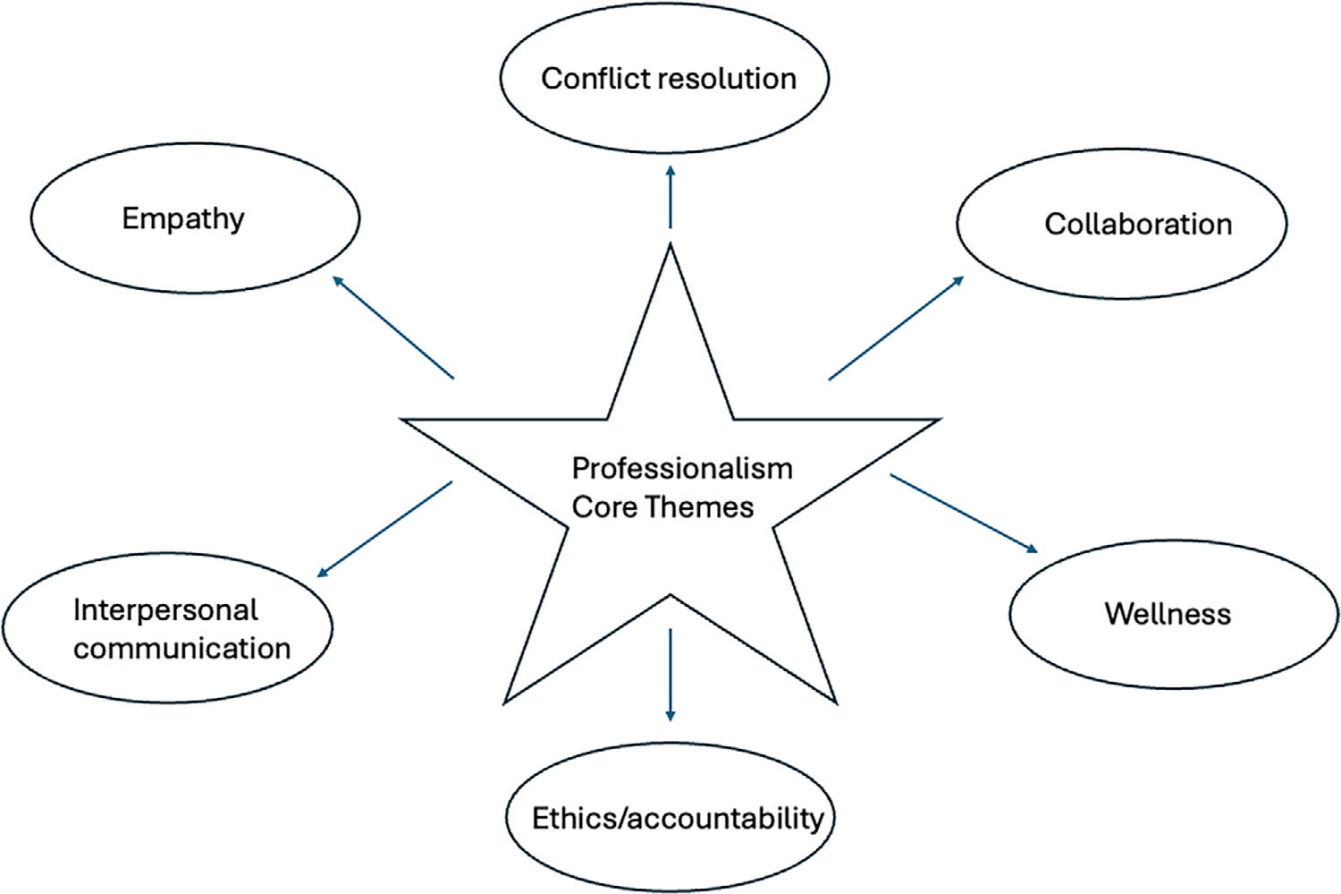
Core themes identified through the evidence‐based review.

Based on the search results, a 12‐month longitudinal curriculum was devised centered on the core themes that emerged (Figure 2). The 537 peer‐reviewed publications chosen to develop this thematic framework could be broadly categorized as follows: ethics/accountability (*N* = 131); conflict resolution (*N* = 120); collaboration (*N* = 107); interpersonal communication (*N* = 70); empathy (*N* = 57); and wellness (*N* = 52). Recognizing that overlap between the themes was subjective and considerable, the six themes were then further stratified into distinctive modules which could be used for formative teaching purposes. The 12 titles that were devised were: (1) Kindness; (2) Mindfulness and emotional intelligence; (3) Difficult conversations and effective communication; (4) Bullying and anti‐bullying; (5) Conflict resolution; (6) Culture of safety and mistake reporting; (7) Burnout; (8) Teamwork; (9) Equity, diversity, and inclusion; (10) Microaggressions; (11) Trust and reliability; and (12) Workforce engagement and empowerment.

The goal of the curriculum was to develop a framework comprised of interactive sessions encompassing each of the core professionalism themes and to devise monthly discussions based on selectively chosen peer‐reviewed articles that represented each theme. To recognize the influence of social constructivism in learning and to emphasize a collaborative approach to promoting professionalism awareness, the curriculum was intentionally designed to instigate collective discussion among people from diverse backgrounds and occupations. A particular emphasis was placed on inner reflection, including the sharing of personal experiences and anecdotal lessons.

### Implementation

**FIGURE 3:**
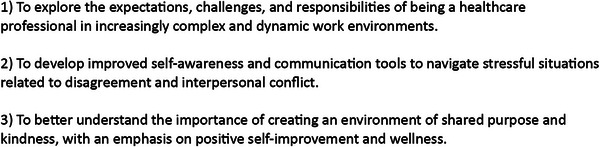
Learner objectives.

The professionalism curriculum was initiated in November 2023 and was designed to run over the course of 1 year with each modular session occurring on the first Tuesday of the month at noon. Table 1 outlines the final professionalism curriculum and detailed list of titles. Monthly sessions were designed to occur via videoconference, and invitations were sent electronically to approximately 50 trainees, faculty, and staff across the institution. Participation was voluntary although moderators were typically assigned 1 month in advance and were given instructions to stimulate discussion. These leads were selectively chosen so that representation would be equal across all segments of the workforce including managers, faculty, physicians, trainees, nurses, and other ancillary personnel. Frequently, moderators were solicited to compile a list of thought‐provoking questions in advance of the actual session. Formal slideshow presentations were not required but often performed at the discretion of the individual moderator. Verbiage used in the electronic invitation, which was typically sent 2–3 weeks prior to the date of the session, was deliberately designed to reinforce the open and non‐threatening nature of the learning format, which consisted of “interactive discussions, simulation scenarios, literature review, and self‐reflection—with the goal of promoting awareness of how professionalism influences the healthcare setting.” An additional reminder was sent approximately 1 day before the session. To promote asynchronous, self‐paced learning and to emphasize the practical applications of the curriculum, all reading material was distributed 2–3 weeks in advance of each session. Figure 3 outlines the stated learner objectives for the curriculum.

**TABLE 1:** Evidence‐based professionalism curriculum.

Module	Title	Theme	References
1	Introduction: Why kindness matters in the workplace?	Empathy	33,40
2	Mindfulness and emotional intelligence: What can it do?	Empathy	34,54
3	Difficult conversations: Is there an easy way to do this?	Communication	38,40
4	Taming your inner bully: What works?	Empathy	14,24
5	Bystander effect and microaggressions: What is this?	Conflict resolution	35,36
6	Burnout: What strategies are effective?	Wellness	45,47
7	Building teams: What is the most effective strategy?	Collaboration	28,31
8	Equity, diversity, and inclusion in the workplace	Collaboration	52,53
9	Creating a culture of integrity: Why, what, and how?	Ethics/accountability	32,55
10	Conflict resolution: How can we best do this?	Conflict resolution	17,25
11	Trust and reliability in healthcare: Why do they matter?	Ethics/accountability	39,43
12	Workforce engagement: How can this be promoted?	Wellness	15,56

## DISCUSSION

The roles and responsibilities of healthcare organizations in shaping practice, education, and research environments that promote professionalism have been long recognized.^7^ Given the increasingly complex, fast‐paced, and stressful nature of healthcare, the focus on optimizing interpersonal dynamics among the multitude of stakeholders and creating a culture based on respect, trustworthiness, and empathy is unquestioned. The results of the exercise are thus noteworthy because they illustrate the sheer breadth and scope of how professionalism potentially contributes to interactions in the healthcare workplace. Indeed, the most striking finding that emerged from this review related to the multi‐faceted and ubiquitous nature of professionalism. As demonstrated in the core themes that were identified, it was apparent that hardly any aspect of healthcare is not influenced by professionalism. This underscores the importance of thoughtful, evidence‐based training, which engages the entire workforce on a regular basis.

The implications of professionalism training with respect to risk management are intuitive. Numerous studies have shown that professionalism lapses are a major contributor to medical malpractice lawsuits from patients and are frequently distinct from quality of care.^8^, ^9^ Additionally, a culture that permits unprofessionalism to go unaddressed can increase the risk of workplace violence and/or employment‐related grievances.^10^ The effect of professionalism on clinical quality and safety including the incidence of adverse events has also been well‐established.^11^ Thus, the fostering of cohesive teams not only protect patients from risks and improve outcomes—they also create a more positive, engaging, and resilient work environment. Indeed, organizations in which staff report higher levels of teamwork have been consistently shown to have lower rates of workplace injuries, truancy, harassment, and bullying which have translated into higher rates of employee retention.^12^

Yet studies have consistently shown that providers have differing perspectives and understanding of professionalism.^1^, ^2^, ^3^ Chan et al. utilized a series of semi‐structured interviews among various providers on conduct, communication, competency, collaboration, and image to demonstrate that variability existed with respect to how individuals view the importance of some facets over others.^13^ Sibbald et al. conducted a qualitative study interviewing faculty and trainees on the subject of professionalism lapses. Among the themes that emerged was that professionalism is highly subject to the interpretation of individuals, and lapses are commonly “in the eye of the beholder” with definitions varying with institutional and situational norms in many cases.^14^ Bernabeo et al. employed physician interviews and a grounded theory approach to better understand provider perceptions.^15^ They concluded that since physicians conceptualize professionalism as a dynamic, subjective competency, opportunities for continual and lifelong learning should be prioritized. Similarly, Shaull et al. used a series of semi‐structured interviews with first‐generation medical students, residents, and physicians to emphasize the need to involve diverse voices in refining professionalism definitions given the evolving nature of this concept.^16^

Given the relative lack of agreement on the definition of professionalism as well as the dearth of literature related to curriculum development on this subject, the need to engage in thoughtful planning for educational purposes should be highlighted. In this sense, the development of the all‐encompassing, longitudinal curriculum presented herein serves as a practical framework for thinking about how to present this topic with the goal of engaging the healthcare workforce.^17^, ^18^, ^19^ Importantly, the evidence‐based curriculum that was developed for learners was designed not just for trainees but also for faculty and staff. This is particularly relevant since residents have been shown to rate their commitment and that of the residency to professionalism higher than that of the institution.^20^ Indeed, in one study, residents reported that only 25% of faculty modeled ideal behaviors all the time; and more than half stated poor role modeling impacted their attitudes about the importance of professionalism.^21^

Despite the increased recognition of the importance of professionalism in healthcare, formal initiatives to promote awareness remain relatively lacking, and a variety of impediments exist which make the adoption and implementation of such programs difficult. Given the lack of cohesiveness around the definition of professionalism, it is not surprising that only a small proportion of residency programs have integrated formal training on this core competency into their teaching curriculum.^22^ Similar to the present study, Valenziano et al. developed a modular‐based curriculum for early practitioners delivering training in five core skill areas: listening for meaning, soliciting another's perspective, negotiating a transparent plan of care, attending to nonverbal communication and microaggression, and speaking up the hierarchy.^23^ Participants reported that the program helped them gain an understanding of each other's roles and workflow challenges, which positively allowed for the cultivation of professional relationships outside the clinical environment thus promoting collegiality and empathy. To provide a practical framework for addressing deficiencies and remediating unprofessional behavior, Barnhoorn et al. recently proposed a qualitative, multi‐level model focused on environment (“where am I?”), behavior (“what am I doing?”), competencies (“what can I do?”), beliefs and values (“what do I believe in?”), identity (“who am I?”), and mission (“why do I do what I do?”).^24^

The use of simulated environments based on real‐life issues encountered in the workplace can also serve as an effective basis for continued development of professionalism among the workforces. Conran et al. developed a series of case vignettes based on actual scenarios pertaining to the domains of service, research, and education while incorporating the principles of duty, integrity, and respect.^25^ General and specific questions pertaining to each case were generated to reinforce model behavior and overcome professionalism issues. Mianehsaz et al. similarly devised a curriculum based on 12 scenarios designed to simulate professionalism challenges.^26^ The investigators showed significant improvements in the participants’ understanding of professionalism upon completion of the training based on self‐rating.

While the goal of the present research was to identify stand‐alone core subject matters that could be methodically used to develop this professionalism curriculum, it must be noted that significant overlap existed between the themes. From a practical standpoint, the emphasis on civility is especially instructive. This is because as society continues to grow in diversity, the importance of creating a sense of belonging in the workplace is paramount. In this sense, acknowledging that interpersonal disagreements invariably arise in the workplace is the first step to prioritizing the teaching of conflict resolution. This relates to the central role of inclusiveness in promoting a healthy work environment where all stakeholders are respected and valued. Indeed, research on group dynamics continue to show that incivility, including rudeness, overt intimidation, aggression, humiliation, and uncooperative or passive‐aggressive behavior, contributes to employee dissatisfaction, turnover, and errors across a variety of industries.^27^, ^28^, ^29^, ^30^, ^31^, ^32^, ^33^, ^34^

The importance of creating a culture where individuals feel comfortable reporting transgressions has also been increasingly recognized.^35^, ^36^, ^37^ A survey of managers found that 13% of their time is spent resolving conflicts arising from rude or uncivil behavior, the equivalent of more than 6 weeks per year.^38^ As such, a significant proportion of the data reviewed makes it clear that delivery of healthcare is increasingly defined by teams of professionals who need to communicate well, respecting the principles of honesty, respect for others, and accountability for their actions.^39^, ^40^, ^41^, ^42^, ^43^ Relatedly, provider well‐being is essential to their expression of their professionalism and capacity to provide compassionate and effective patient care.^44^ In this regard, evaluating the role of burnout is an essential component of any professionalism curriculum.^45^, ^46^, ^47^

The limitations of this study relate to the inherent problems in uniformly defining professionalism in the healthcare setting. While the feasibility of developing a framework for professionalism education based on the evidence‐based review was demonstrated, it must be acknowledged that a considerable degree of subjectivity was involved in the categorization of papers, which varied by design, methods, and inclusion criteria. It was also not possible to account for areas of overlap between the themes. Similarly, it was difficult to account for the role of cultural bias in influencing professionalism. Rees et al. showed in global health clinic that that behaviors that may be considered best practice in one clinical setting may be perceived as unprofessional in another.^48^ Additionally, variations in professionalism particularly with respect to what constitutes acceptable behavior exists across specialties and vocations in healthcare.^49^ The impact of digital health in contributing to professionalism also needs continued exploration.^50^ For instance, with the advent of electronic communication, the potential to introduce new breaches in etiquette and civility has been shown.^51^

It must be acknowledged that this study did not present any quantitative data but instead only offered a descriptive synthesis of the available literature with the goal of formative curriculum development. As such, one of the primary limitations was that the presented framework was devised as an interpretive construct of the author and subject to unforeseen biases. Furthermore, the absence of objective learner‐rated data precludes an assessment of how the curriculum may have affected the opinions and attitudes of individuals. Future research will involve the analysis of learner‐reported metrics related to behavioral change and lessons learned. Additionally, the impact of such a curriculum on organizational culture as measured using a variety of clinical, medico‐legal, workforce, and patient‐reported benchmarks requires investigation so that educational processes can be refined over time.

Lastly, because many of the core themes identified herein can be emotional subjects, creating a safe and dedicated environment where people are encouraged to discuss these openly in an inclusive fashion is essential.^52^, ^53^ For those working in medicine, many of whom are simply trying to get through the many responsibilities of the workday, this is particularly true; and in some sense, there continues to be sentiment that sharing and talking about personal issues is not appropriate for the workplace. This again underscores the importance of taking a deliberate, evidence‐based approach to promoting curriculum design with the goal of workforce engagement– with the goal of delicately bringing the subject of workplace culture to the forefront. To promote effective “buy in,” visible, sustained, and proactive participation from leaders across the institution will be imperative for future success. Ultimately, the ability to transition from curriculum development and a framework for professionalism training to tangible benefits in terms of positive cultural change will require consistent support from organizational leadership at all levels.

## CONCLUSION

The development of a thematic‐based curriculum, which was rooted in an evidence‐based, methodical literature search was shown to be feasible with the goal of fostering critical discussion around a variety of professionalism issues germane to healthcare. Given the vastness of content that must be considered, the proposed framework for interpreting the broad amount of disparate data and varying perspectives on professionalism might be of utility to effectively teach and engage the workforce. Since professionalism has been shown to permeate nearly every aspect involved in healthcare workflow, the potential impact of educational initiatives is immense. Ultimately, a high standard of professionalism benefits all stakeholders as it sets a realistic benchmark for conduct and for self‐awareness across an entire community. Continued and ongoing refinement of the principles brought out in this study will be required as definitions of professionalism evolve in the future.

## CONFLICT OF INTEREST STATEMENT

The author was awarded the 2022 John Wooden Leadership Fellowship by the University of California, Los Angeles‐ Anderson School of Management in partial recognition of this original work.

## Data Availability

There is no original data arising from this work.
